# Implant and submodeling techniques for detailed finite element study of inserts in composites

**DOI:** 10.1016/j.mex.2019.09.012

**Published:** 2019-09-13

**Authors:** Venkateswarlu Gattineni, Venukumar Nathi

**Affiliations:** Department of Mechanical Engineering, GITAM School of Technology, Hyderabad, India

**Keywords:** Detailed study of structural implants, Implant, Submodeling, Smeared, Contagious, Rebar, Interpolation, Stiffness, Host, Guest

## Abstract

Plastics and other specialty materials such as composites and concrete etc. are effectively used under situations which require less weight with more load bearing capacity. Based on specific design requirements, the parent material with which the component is primarily made of, is inserted with implants, whose size could be very small compared to the over all component/system. These inserts could serve multiple purposes such as providing localized stiffness, provide improved strength and stiffness for attaching other components, facilitate wiring harness, Allow liquidous circuit through pipes etc. When used as a stiffener for strength enhancement, the contribution of the implant needs to be studied in detail using techniques such as finite element method to evaluate its contribution besides examining it for any localized failure in a detailed manner. Different commercial software has different capabilities and hence different methods are followed by engineers across multiple disciplines to perform detailed study of these inserts. Each of such methods have their own advantages and disadvantages. This study proposes a generic algorithm in conjunction with sub modeling technique using finite element methods, which can be used with any of the commercial finite element software for improved and detailed study of the implant behavior/contribution.

• The proposed method combines the advantages of the of existing approximate methods such as embedded and 3D composite methods
• The proposed method consists of 2 linear finite element computations in which the first gives approximate results for simplified geometry, while the second calculation gives accurate and finer results using submodeling technique
• The method can be generalized to be applied for any complex shaped implant geometries with multiple materials

## Graphical abstract

**Figure:**
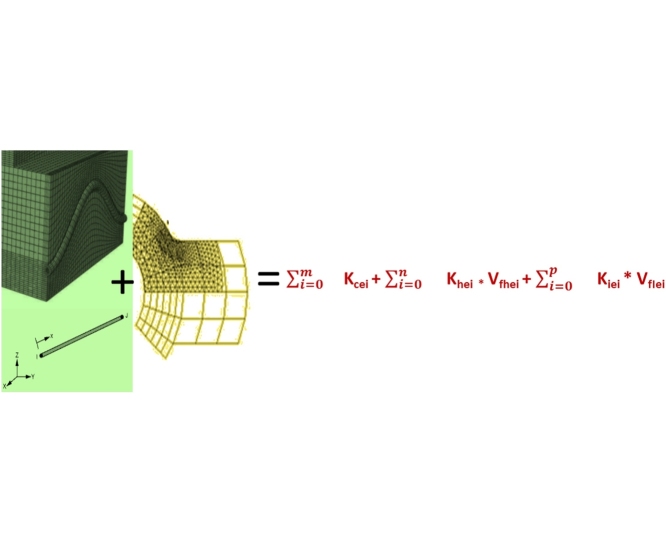

**Specification Table**

**Table:** 

Subject Area:	*Engineering (Solid Mechanics using finite element techniques)*
More specific subject area:	*Methodology or algorithm that can be applied for a detailed implant study using finite element based structural study*
Method name:	*Detailed study of structural implants*
Name and reference of original method:	*Embedded element techniques* [2] *and composite modeling with rebar options* [1]
Resource availability:	*Multiple scholarly articles dealing with designs, which have implants inserted into a component made of different material.*

## Method details

### Background

In this study a new method namely, “*Implant and submodeling techniques for detailed finite element study of inserts in composites*” is proposed for detailed finite element study of Inserts in composites.

Majority of the present-day designs contain a large component into which an implant (a small component made of different material than the large component) is inserted for strength or functional purposes. Such practices are found frequently in the domains of Automotive (For tire behavior modeling studies – Steel wires inserted in rubber), Biomechanics (Injury modeling where bones are treated as implants inside the flesh-matrix), Industrial (Where stiffeners pass through foam filled structures) and Civil cngineering (Concrete matrix reinforced with steel bars as implants). Different engineers use different practices for structural behavior study of such designs based on commercial finite element software used. One of the widely used, but time-consuming method (Both for finite element modeling and computation) considers modeling the large component as a solid with cavity filled with solid equivalent to the implant. The mating surfaces of the implant and the large component usually have contagious finite element meshes which are normally connected using rigid connections. If contagious mesh pattern generation is an issue, the mating surfaces are usually tied using contact regions which are treated as fully bonded [1,2]. These methods which is generic, consume lot of time in geometric modeling, finite element mesh generation and connection/contact definition creation. If contagious mesh with rigid connections are used, it increases the problem size in terms of degrees of freedom and some ill conditioning of the stiffness matrix (Due to sudden and high stiffness at interface regions as a result of rigid connections). If bonded contact methodology with non-conforming mesh at matrix implant interface is used, it leads to nonlinear computations resulting in very high solution time. Using either of these methods, the behavior of the large component and implant can be captured realistically, but with higher efforts for modeling and/or computation time. But a practical design exercise involves several design iterations and computations and hence there is need for simplified methods which requires less time while being close to accurate, some of which are available in commercial finite element software [1,2] and are described in later sections.

### Embedded element method

**Fig. 1:**
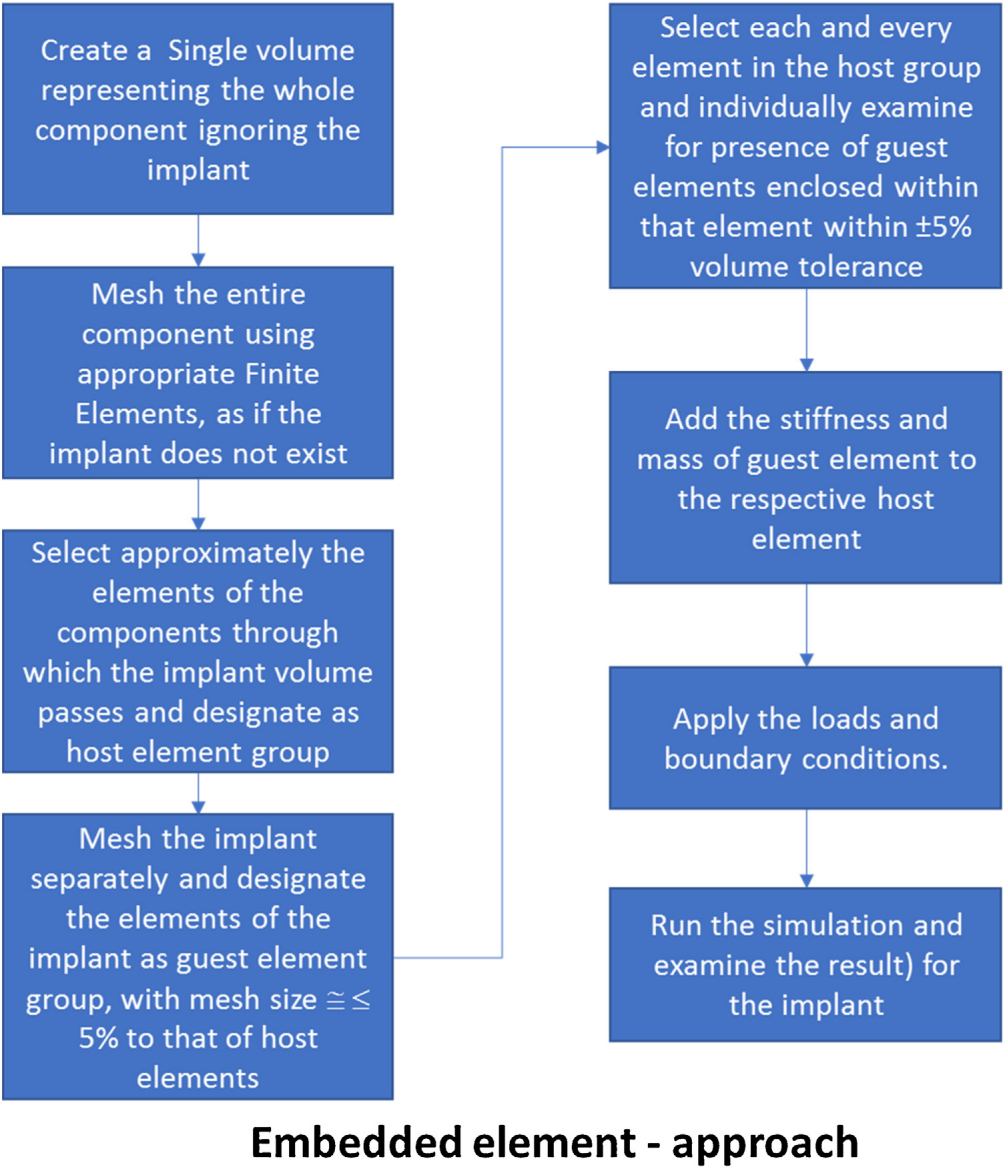
Process chart for the embedded element approach.

The embedded element capability available in Abaqus [2,3,6] allows the two different materials (Implant and the matrix material holding it) to be meshed using finite elements independently, with no connectivity. The matrix material holding the implant is modeled as if there is no insert in it. The implant is meshed independently (Considering almost same mesh size parameters) and a contagious mesh discretization and establishment of connectivity is not required. Hence the effort required to make an acceptable mesh is drastically reduced. In this methodology, the finite elements of the matrix in the region where the implant elements lie in space are specified as host elements. During the process of the solution, the software searches for the implant elements (That are interchangeably referred to as guest elements in later sections) that are fully enclosed inside a host element within the given tolerance. The stiffness and mass of such implant elements is added to the stiffness and mass of the corresponding host element. Hence in this method, the stiffness and mass of the host element is increased, and the computation is performed. The solution phase begins after the stiffness and mass modifications. During this phase, the mesh of the implant elements is ignored for the time being. After the computation is performed, the averaged or smeared results are super imposed on the implant mesh for postprocessing or study purpose. This method works very well if one of the materials(s) (Host or implant) has very less density (Such as steel and foam combination) compared to the other. But if the densities of the host and implant materials are comparable, the simple addition technique would give rise to increased stiffness and mass of the overall structure. Hence this method is predominantly used wherever the stiffness of the implant is expected to be significantly higher than that of the host (Matrix) elements holding it. Fig. 1 describes the proposed process in the form sequence of operations.

### D composite element method

**Fig. 2:**
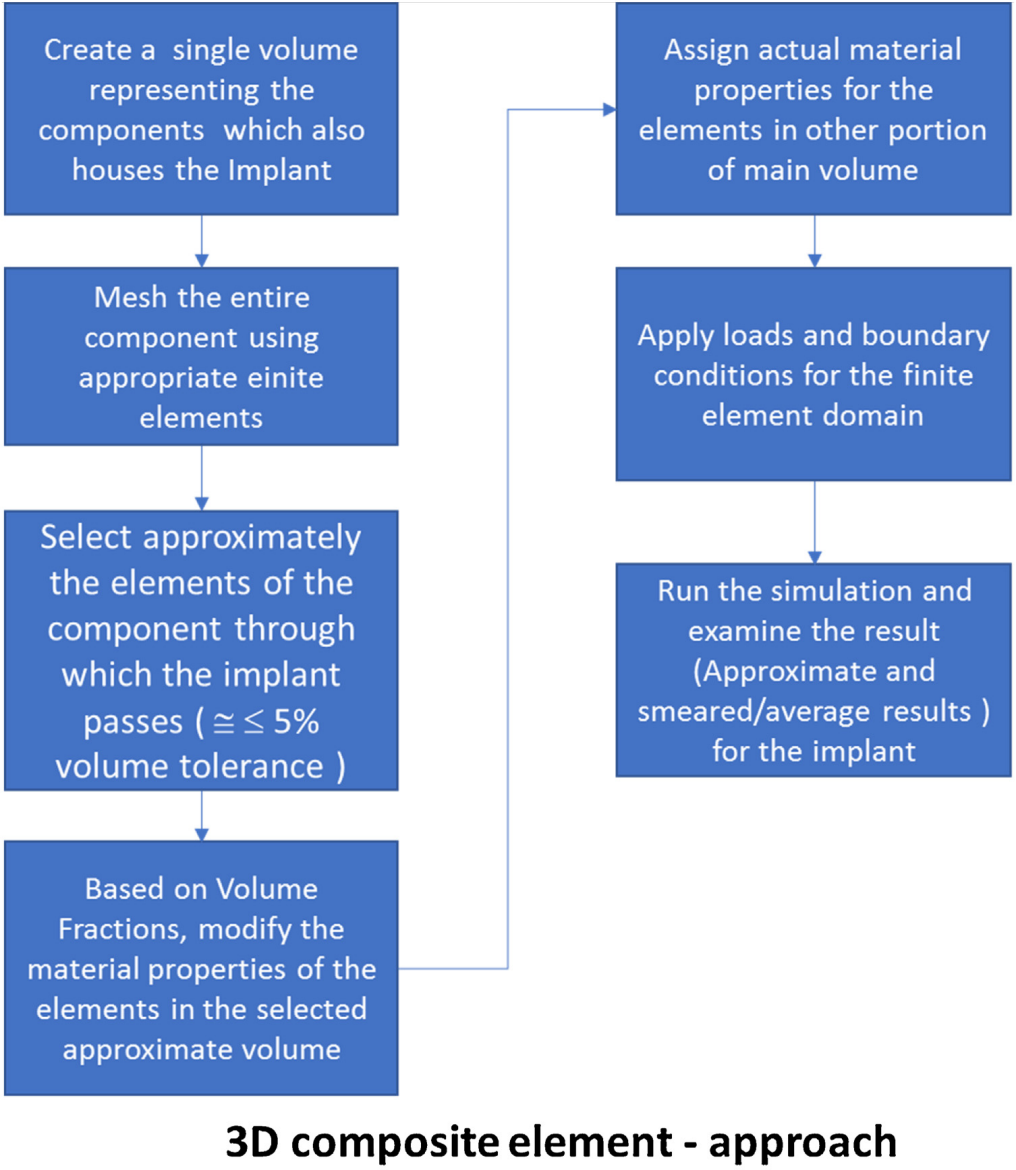
Process chart for the 3D composite element approach.

The composite element capability available in Ansys [1,4,5,7] requires Finite Element modeling of the only host elements (Matrix). But additional information about the implants such a material property and the volume fraction of the implant material along the host element direction needs to be specified in advance. Hence during the solution, Ansys considers the stiffness and mass contributions (based on volume fraction) for the implant volume regions and performs the calculations. The user also has the option to specify few locations in 3D space on the implant, where the detailed results are required (Stress, Strain, Displacement etc..). After the solution is complete, Ansys software interpolates and outputs the results at previously requested locations for study. Hence the behavior for the implant is available only at few select locations in text form. In this approach there are no errors introduced in the form of additional stiffness or mass. But the results are of average value. To avoid loss of accuracy due to averages, one must ensure that the mesh size is as small as possible. Fig. 2 describes the proposed process in the form sequence of operations.

### Submodeling

**Fig. 3:**
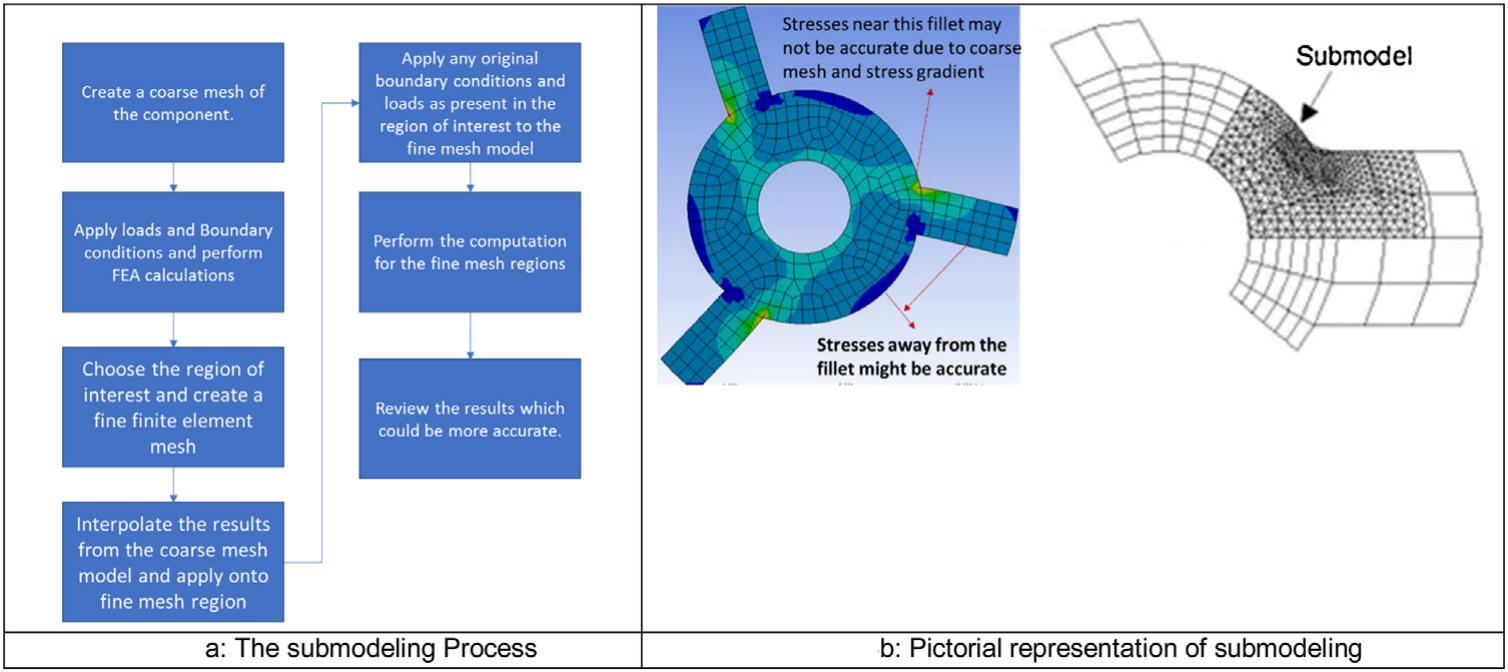
a. The submodeling Process. B. Pictorial representation of submodeling.

The submodeling [1,2] process is based on St. Venant’s principle which states, “The difference between the effects of two different but statically equivalent loads becomes very small at sufficiently large distances from load”. The concept is based on the theory of creation of coarse finite element mesh for the component or assembly. It is assumed that the small features of the geometry do not have the impact on the overall response of the system and are ignored for mesh discretization. The primary hypothesis is that the local stiffness does not have influence on the strain beyond that local region. After the structure with coarse mesh is used for computation using finite element method, a very refined finite element model is made which is called as the submodel, for the region of interest. The results from the coarse mesh (usually displacement for structural analysis) are mapped through interpolation based on element shape function onto the sub model nodes as boundary conditions. The original loads if any in the region of interest are also re-applied and the finite element model is rerun to get accurate and refined response, in the area of interest. Fig. 3a and b describes the submodeling process step by step and pictorially.

### The proposed approach

**Fig. 4:**
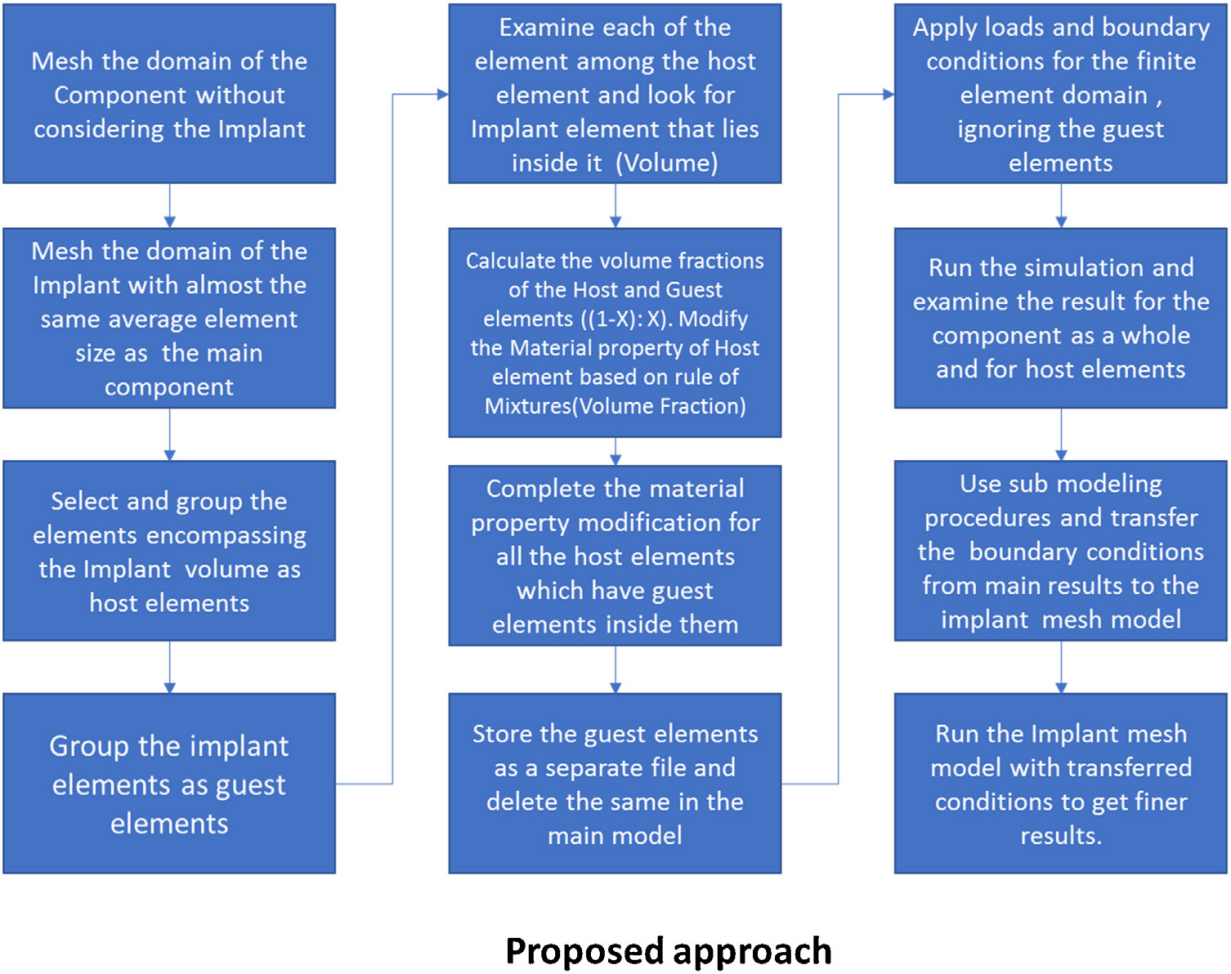
Process chart for the proposed/improved approach.

The proposed approach combines the advantages of the above 3 methods (Embedded element method, Composite Element, Submodeling). In this method, First the matrix or the component is discretized using the finite element ignoring the implant. Then the implant is also discretized using similar finite elements (Shape). The size of the implant elements can be slightly lesser than those of matrix elements (1–5%) This leads to overlap of the elements of matrix and implant. The finite elements of the matrix which overlap or enclose those of the implant are chosen as host elements. Within each of the host elements, a search is made (Within the specified volume tolerance) to find out the enclosed element or elements of the implant (Guest elements). This process is similar to the embedded element concept [2]. Now, the material property of the corresponding host element is modified as per the volume fractions of the host and the implant element [1], which is like the treatment for 3D composite element. Whole of this process (Search for implant elements and modification of the material properties based on volume fractions) can be automated using scripts (Python [2] and APDL [1]). Once this is done, the implant elements are exported to a separate file and deleted. Then the computation is done to get the structural response for the entire component. The results thus obtained shall be the combined average results for the host and implant elements at the region of implant. Then using submodeling approach [1,2], the displacement results are interpolated and imposed onto the nodes of the implant mesh model and computation is done to get finer response for the implant, with actual implant material properties. This way, 2 linear Finite Element studies are performed. The need for a contagious mesh or contacts behavior modeling is eliminated, besides avoiding error in terms of stiffness and mass addition. The submodeling calculation can also be performed for the implant with much more refined mesh for more accuracy. Fig. 4 describes the proposed process in the form sequence of operations.

#### Assumptions and guidelines for the proposed approach

• The major assumption for the proposed approach is that there is always perfect bonding between the Matrix and implant materials even when the load is acting.
• The host elements have always a 3D shape (Brick or pyramid or tetrahedron)
• The guest (Implant) elements can be Brick or tetrahedron or pyramid or 3D shell or 3D beam as permitted by the respective CAE software
• Rotational degrees of freedom if any for the guest (Implant) elements are ignored
• The mesh size of the guest (Implant) element shall be always equal to or less than those of the host elements with a small tolerance. Even the size of the guest elements can be multiples of ½ of the size of the host elements
• There can be multiple guest (Implant)elements in a host element that too with different material properties for each
• The geometry of the Implant shape can be simplified by removing fine features such as fillets, notches drafts etc.… while generating mesh. However, for the computation or back substitution using submodeling method, a different mesh which is much finer in size and has all the small features of implant such as fillets can be included for obtaining detailed results.
• After identification and categorization of mesh as host and guest elements, the entire process can be automated using scripting capability/option available in the respective CAE software [1,2]
• No loads and boundary conditions are applied to the nodes and elements of guest elements

#### Qualitative comparison of the methods in practice and proposed/improvised method

The qualitative comparison of the existing methods which are being used with that of the proposed method is shown in Table 1.

**Table 1:** Qualitative comparison of existing and Proposed Methods.

	Type of Finite Element Mesh	Connections	Solution Type	Degree of Freedom	Modeling Time	Computation time	Results of the Implant
1	Contagious mesh	Rigid	Linear	Medium	High	Medium	Accurate
2	Non contagious mesh	Contact Surface	Non-Linear	High	High	High	Accurate
3	Embedded element	None	Linear	Low	Low	Low	Additional Stiffness and Mass
4	3D composite element	None	Linear	Low	Low	Low	Average and smeared
4	Proposed method	None	Linear	Low	Low	Low	Accurate

#### Solid Mechanics of the existing and proposed method(s)

**Fig. 5:**
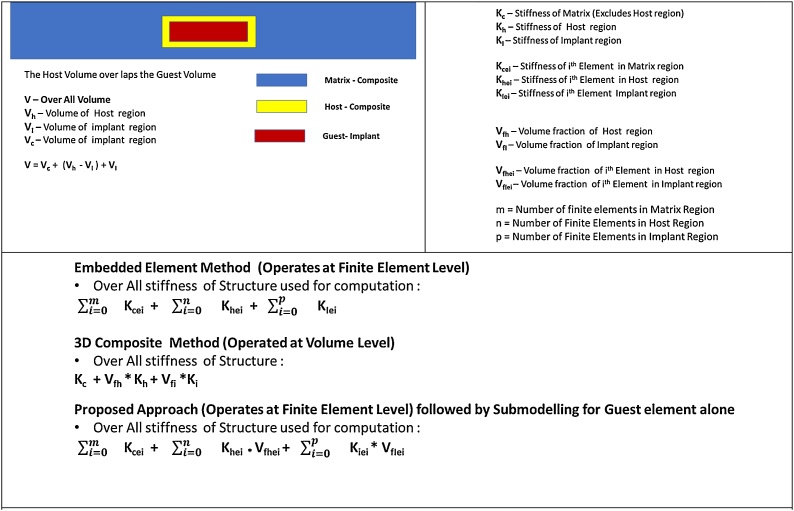
Solid Mechanics of the existing and proposed methods.

Fig. 5 Describes the mechanics of the present and proposed methods

#### Pseudo script of the proposed approach

**Figure:**
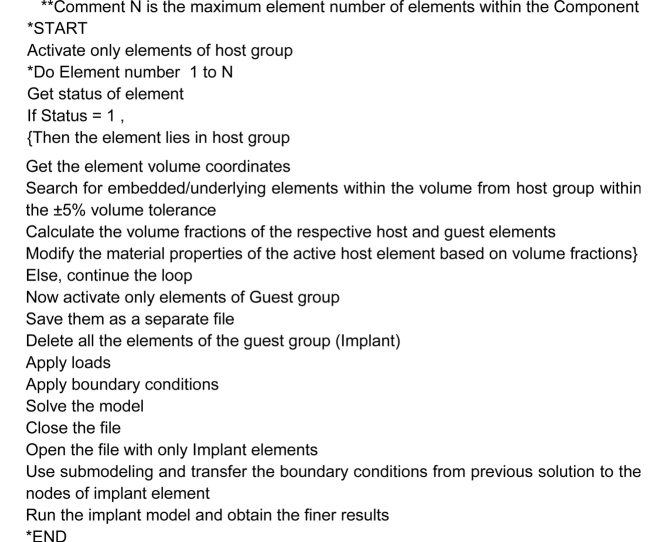

##### Material properties of the main component/matrix

The following are the material properties used for the matrix (Derived from tests as per ASTM) used for the calculations [8].

**Table:** 

Elastic Material Properties (Anisotropic)								
Young’s modulus			Shear modulus			Poison ratio		
E_11_(MPa)	E_22_(MPa)	E_33_(MPa)	G_12_(MPa)	G_23_(MPa)	G_31_(MPa)	Ѵ_12_	Ѵ_23_	Ѵ_31_
58093	58093	9759	3545	2564	2564	0.0154	0.5356	0.1575

##### Material properties for the implant

For the sake of demonstration, the material of the implant has been assumed to be aluminum with the following material properties.

Young’s modulus (E): 70,000 MPa

Poisson ratio: 0.28

##### Geometry, load and boundary conditions

**Fig. 6:**
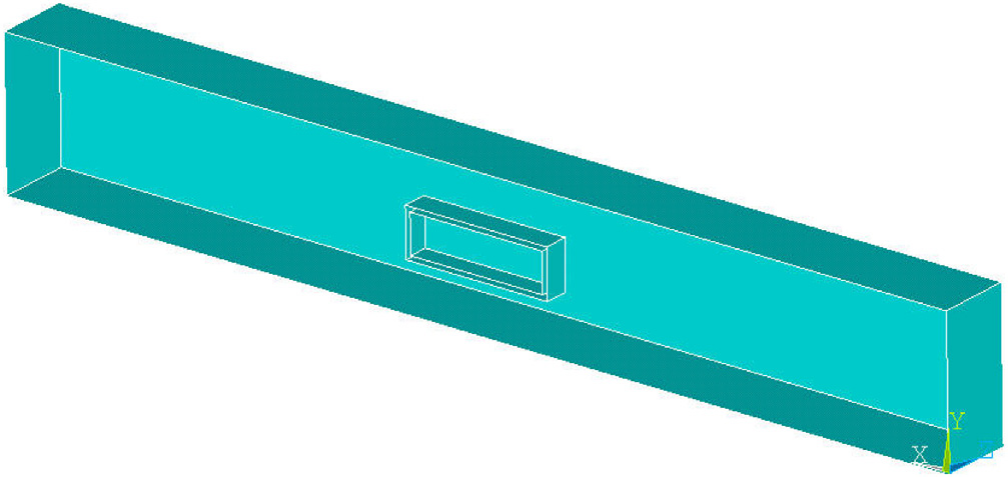
Volume blocks used for the method demonstration.

For the sake of the study a simple cuboid of dimension 200 × 30 x 30 mm is chosen as the main component (Matrix) and it is modelled as a cantilever beam with load at one end and fixed at the other end. The Implant is assumed as an aluminum cuboid of dimensions 28 × 8 x 8 mm and aligns at the geometric center of the Matrix Cuboid. Fig. 6 shows the geometric description of the configuration.

##### Step by step approach

**Fig. 7:**
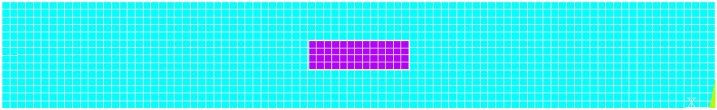
Mesh generated for the study (Implant mesh overlaps with the component mesh).

As shown in Fig. 7, The component is first meshed ignoring the implant geometry and later the implant is also meshed. The implant element mesh overlaps the component mesh.

**Fig. 8:**
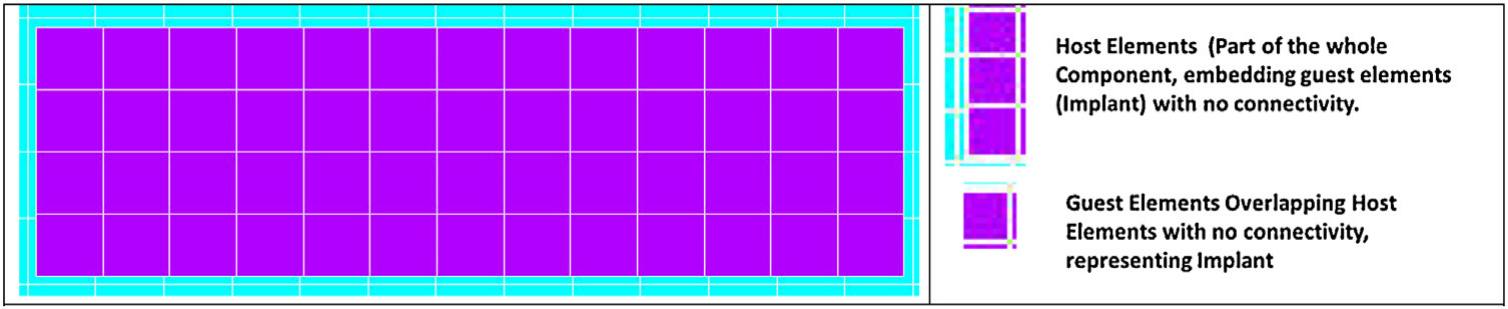
Mesh showing guest Elements overlapping host elements/region.

As shown in Fig. 8, the elements of the component around the implant mesh (Within ± 5% distance) are grouped as host elements, while implant elements are grouped as guest elements.

**Fig. 9:**
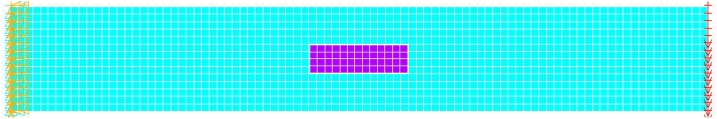
Mesh with applied boundary conditions (Fixed – Left) and loads (Right).

The loads and boundary conditions are applied for the finite element model as shown in Fig. 9.

**Fig. 10:**
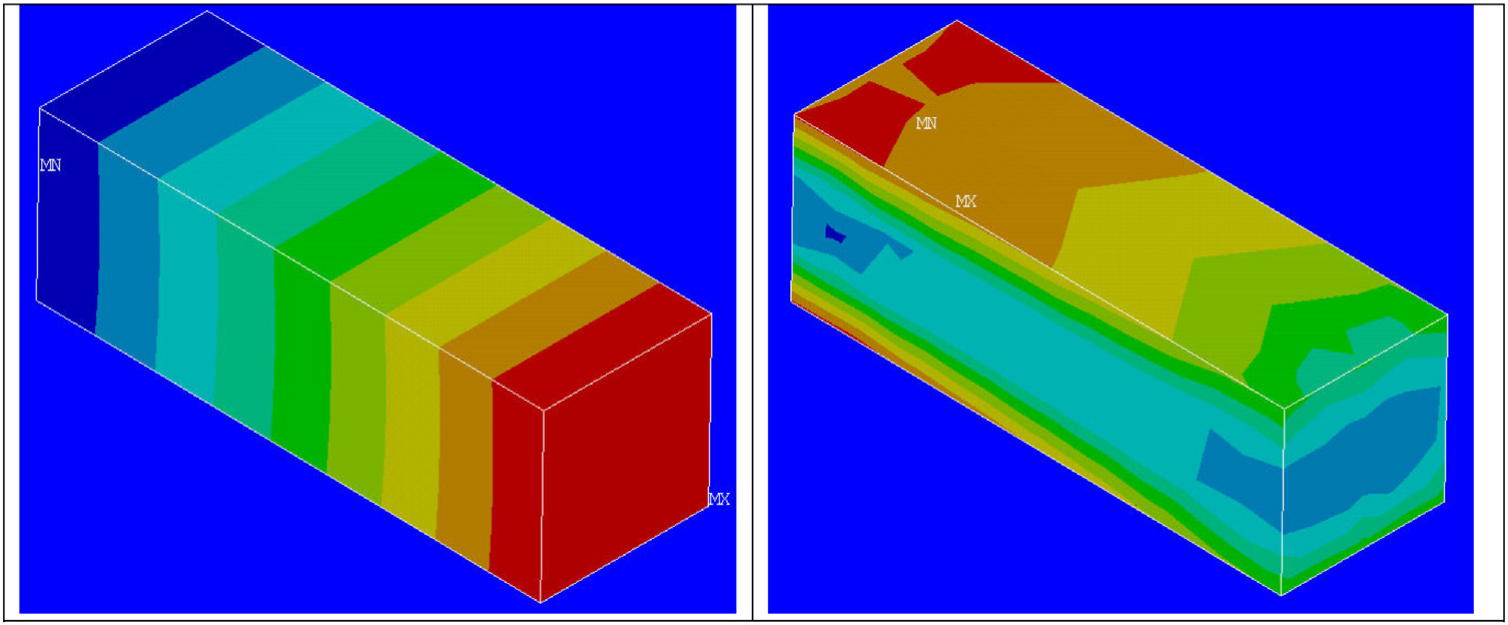
Resultant displacement and equivalent stress plots for host elements after calculations with proposed method.

Later using the scripts based on pseudo script indicated, for each of the elements in the host region, corresponding element from the implant is identified. Based on respective volume fractions of the elements in host and guest regions, the material properties of the individual element in host region is modified [9] and the calculations are done. Fig. 10 shows the results of calculation for the host elements.

**Fig. 11:**
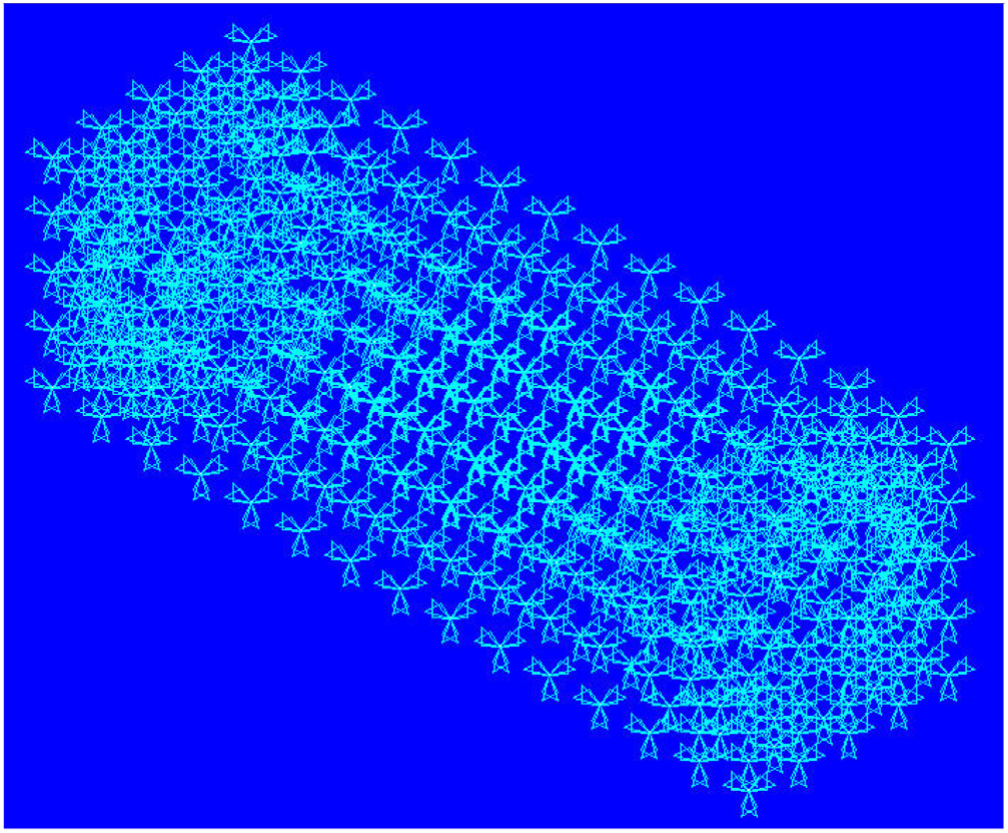
Submodeling output transferred as boundary conditions to the implant mesh.

Based on sub modeling technique, the results are transferred to the nodes of the implant mesh as shown in Fig. 11. The implant mesh is assigned its original material properties and the computation is done (Second linear Finite element analysis – For Implant alone).

**Fig. 12:**
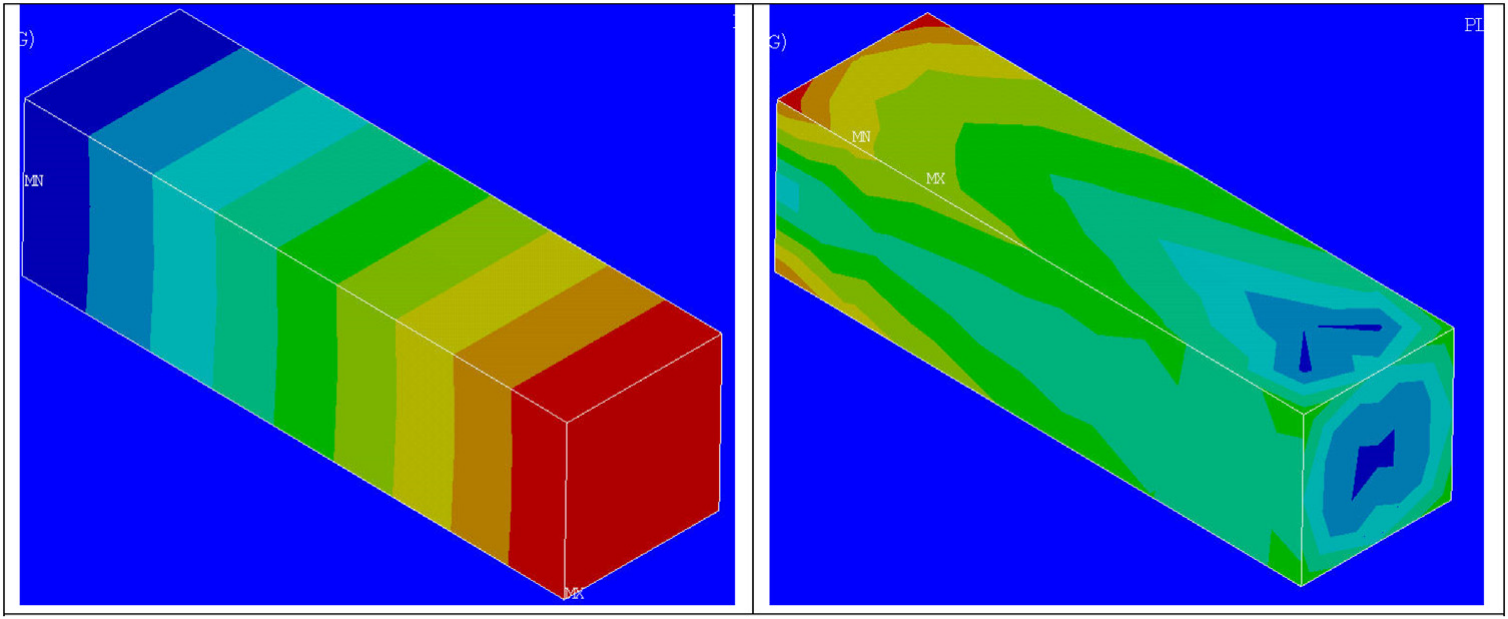
Resultant displacement and equivalent stress plots for implant after calculations based on submodeling.

The finer results for the implant after the calculations are shown in Fig. 12. The comparison between different methods is shown in Table 2.

**Table 2:** Results comparison for existing and proposed methods.

	Type of finite Element Mesh	Connections	Solution type	Maximum resultant displacement	Equivalent vomnises stress	Results for the implant
1	Contagious mesh	Rigid	Linear	0.104	2.863	Realistic
2	Non-Contagious mesh	Contact Surface	Non-Linear	0.104	2.863	Realistic
3	Embedded element	None	Linear	0.102	2.949	Additional stiffness and mass
4	3D composite element	None	Linear	0.100	2.927	Average and smeared
4	Proposed method	None	Linear	0.104	2.863	Close to realistic

## Discussion on method and result

For the sake of demonstration, the process has been executed using simple geometry, loads and boundary conditions. However, in practice, the actual geometry, loads and boundary conditions could be complex. Also, in the current example, a single implant made of single isotropic material has been chosen. In Practical scenarios, implants made of different and anisotropic materials could exist. The proposed method is a two-pass method where in the solution is first made for the component and then the conditions are transferred to the model of Implant using sub modeling techniques.

## Conclusions

• The proposed methodology eliminates the need for connectivity between finite elements of insert and matrix components there by saving valuable preprocessing time. Higher the number of inserts inside the matrix, higher shall be the saving in preprocessing time.
• The methodology provides a faster solution with equivalent accuracy for global stiffness, for assemblies of composite matrix with inserts made of different materials.
• A simplified implant model can be used during the component analysis ignoring small features like notches and fillets for the first calculations. The finer details like notches, fillets etc. can be included with a fine mesh for the submodel and finer results can be obtained at stress concentrations zones.
• The proposed methods also eliminate the need for contact modeling in between the insert and matrix there by saving lot of solution time. Hence within available time more design iterations can be performed to obtain an optimal design.
• The proposed method combines the advantages of the embedded element method [2], 3D composite method [1] and submodeling [1,2] to give accurate results.
• The methodology can be easily extended to study multiple inserts inside a matrix made of multiple materials passing through host element region, considering the multiple volume fractions of the respective materials.
• The methodology can also be extended to designs experiencing dynamic loads. In such cases, the density of the elements in the host region needs to be altered based on the volume fractions.

## References

[1] Ansys Theory Manual, Version 19.0.

[2] Abaqus Theory Manual Version 2019.

[3] Garimella H.T., Menghani R.R., Gerber J.I. (2018). Ann. Biomed. Eng..

[4] Joosten M.W., Wang C.H., Mouritz A., Khatibi A.A., Agius S., Dingle M., Trippit B., Cox B. (2014). Application of the embedded element technique to predict interlaminar failure. ASC 2014: Proceedings of the American Society for Composites 29th Technical Conference, DEStech Publications.

[5] Gebreyohaness A.S., Clifton G.C., Butterworth J.W. (2012). Finite element modeling of non-ductile RC walls. Proceedings of the Fifteenth World Conference on Earth Quake Engineering.

[6] Matveeva Anna, Böhm Helmut, Grygoriy Kravchenko, van Hattum Ferrie (2014). Investigation of the embedded element technique for modelling wavy CNT composites. CMC: Comput. Mater. Continua.

[7] Hamid Nubailah Abd, Ismail Muhammad Hussain, Ibrahim Azmi, Adnan Azlan (2018). Finite element analysis of smart reinforced concrete beam with super elastic shape memory alloy subjected to static loading for seismic mitigation. AIP Conference Proceedings 1958.

[8] Derewonko Agnieszka, Gieleta Roman (2012). Carbon-epoxy composite fatigue strength – experiment and fem numerical estimation. J. KONES Powertrain Transp..

[9] R.P.L. Nijssen, Composite Materials an Introduction, 1st English Edition, Based on 3rd Dutch Edition (ISBN: 978-90-77812-51-8 Copyright©2015 R.P.L, ISBN: 978-90-77812-471, Inholland University of Applied Sciences, Nijssen. www.inholland.nl/lectoraatgrootcomposiet.

